# Alzheimer’s Disease and Its Association With Bone Health: A Case-Control Study

**DOI:** 10.7759/cureus.13772

**Published:** 2021-03-08

**Authors:** Sameet Kumar, Aakash Chandnani, Norah H Aung, Simra Shahid, Dua Bukhari, Sania Shahzad, Besham Kumar, Sidra Memon

**Affiliations:** 1 Internal Medicine, Chandka Medical College, Karachi, PAK; 2 Internal Medicine, Jinnah Sindh Medical University, Karachi, PAK; 3 Health Sciences, Western Illinois University, Macomb, USA; 4 Internal Medicine, University of Medicine 1, Yangon, MMR; 5 Medicine, Jinnah Sindh Medical University, Karachi, PAK; 6 Internal Medicine, Jinnah Postgraduate Medical Centre, Karachi, PAK

**Keywords:** bone mass density, alzhemier's disease, association

## Abstract

Introduction

Alzheimer’s disease is associated with low bone mineral density. Various studies have linked early-onset Alzheimer's disease with bone health. In this study, we will determine the association between bone health and recently diagnosed Alzheimer's disease in the local population.

Methods

This case-control study was conducted at the neurology unit from April 2019 to Sept 2019. One hundred and fifty (150) Alzheimer’s patients with recently (within the last six months) confirmed diagnoses, based on clinical symptoms, mental status, and computed tomography (CT) scan, were included from the neurology outpatient department. The gender and age-matched 150 healthy participants were included in the study as the reference group. Various parameters of bone health and mental status were measured.

Results

Participants with Alzheimer’s had a significantly lower level of serum vitamin D (15.2 ± 4.2 ng/mL vs. 27.5 ± 8.1 ng/mL, p-value: < 0.0001) and lower level of serum osteocalcin (4.3 ± 1.7 ng/L vs. 5.6 ± 2.0 ng/L, p-value: < 0.0001). Participants with Alzheimer’s disease had more people with T-score ≤ -2.5 as compared to the general population (52.0% vs. 16.6%, p-value <0.0001).

Conclusion

Alzheimer’s disease is associated with poor bone health as compared to the general population of the same age. Bone health can be an important parameter to screen patients at risk of Alzheimer’s disease. The management of Alzheimer’s disease should include a regular assessment of bone health, and the treatment plan should include therapies to improve bone health.

## Introduction

Alzheimer’s disease is an irreversible neurodegenerative disorder that results in progressive cognitive decline. It is caused by an abnormal buildup of misfolded proteins in the brain, which includes neurofibrillary tangles intracellularly and amyloid protein deposits extracellularly [1]. This disease has multifactorial foreplay, with both genetic and environmental factors. Various factors such as advancing age, female gender, and the presence of apolipoprotein E4 have shown to pose an increased risk amongst many others [2]. One of the factors that have been linked to the development of early-onset Alzheimer’s disease is low bone mineral density (BMD) [2]. Various epidemiological studies have shown that Alzheimer’s disease, osteoporosis, and osteopenia happen at the same time in the older population and maybe interlinked [3].

A study conducted in 2005, showed that 11.11% of females in the lowest quartile of femoral neck BMD developed Alzheimer’s disease (AD), whereas 6.0% of females in the other three quartiles developed AD, concluding that low BMD raised the risk of AD in female patients by more than two folds [4]. In a recent study, in 2020, bone metabolic biomarkers were measured in male patients with early-onset AD. They were found to be closely related confirming low bone mineral density as an independent risk factor for the early development of AD [5].

There are no data available demonstrating the prevalence of low bone mineral density in the Alzheimer’s population of Pakistan. In this study, we will compare the BMD, vitamin D levels, serum calcium levels, and serum osteocalcin levels between patients with Alzheimer’s disease and healthy individuals.

## Materials and methods

This case-control study was conducted at the neurology unit from April 2019 to Sept 2019 in a tertiary care hospital in Pakistan. One hundred and fifty (150) Alzheimer’s patients with recently (within the last six months) confirmed diagnoses, based on clinical symptoms, mental status, and computed tomography (CT) scan, were included from the neurology outpatient department. The gender and age-matched 150 healthy participants were included in the study as the reference group. All patients were not taking any sort of vitamin D supplements. Ethical review board approval was taken before enrollment of participants. Informed consent was taken from participants or attendants where the participant was incapable of giving informed consent.

Age, gender, smoking status, body mass index (BMI), and physical activity were noted in a self-structured questionnaire. The Mini-Mental State Examination (MMSE) scale was used to measure cognitive function. After the assessment of patients, blood was taken via phlebotomy and send to the laboratory to assess serum vitamin D levels, serum calcium levels, and serum osteocalcin levels. A bone mass density (BMD) test was done to evaluate the status of bone health. T-score less than or equal to minus one (-1) was considered normal bone destiny, T-score between minus one (-1) to minus two point five (-2.5) was considered as osteopenia, and score more than minus two point five (-2.5) was labeled as having osteoporosis [6].

Statistical analysis was done using the Statistical Package for the Social Sciences (SPSS) v. 23.0 (IBM Corp, Armonk, New York). Continuous variables were analyzed via descriptive statistics and were presented as means and standard deviations (SDs) while categorical variables were presented as frequency and percentages. T-test and Chi-square were applied as appropriate. Relative risk (RR) was used to compare the interventional group with the placebo. A p-value of less than 0.05 meant that there was a significant difference between the two groups and the null hypothesis was void.

## Results

Age, gender, BMI, and smoking status were comparable between both groups. The MMSE was lower in Alzheimer's patients as compared to the reference group (21.4 ± 54.9 vs. 25.9 ± 5.6, p-value; < 0.0001) (Table 1).

**Table 1:** Comparison of Characteristics of Case and Control Group Abbreviation: kg; kilogram, m; meter

Characteristics	Alzheimer’s Group (n=150)	Reference Group (n=150)	P-Value
Age in years (Mean ± SD)	65 ± 10	64 ± 12	Not significant
Male (%)	50	53.3	Not significant
Body mass index greater than 25 kg/m2 (%)	29.3	26.6	Not significant
Current Smokers (%)	10	14	Not significant
Mini-mental Status Examination	21.4 ± 5.9	25.9 ± 5.6	< 0.0001

Participants with Alzheimer’s had a significantly lower level of serum vitamin D (15.2 ± 4.2 ng/mL vs. 27.5 ± 8.1 ng/mL, p-value: < 0.0001) and a lower level of serum osteocalcin (4.3 ± 1.7 ng/L vs. 5.6 ± 2.0 ng/L, p-value: < 0.0001). Participants with Alzheimer’s disease had more people with T-score ≤ -2.5 as compared to the general population (52.0% vs. 16.6%, p-value <0.0001) (Table 2).

**Table 2:** Comparison of Bone Health Status of Case and Control Group Abbreviation: mg; milligram, dL; deciliter, ng; nanogram. L; liter, BMD; bone mineral density

Bone Health	Alzheimer’s Group (n=150)	Reference Group (n=150)	P-Value
Serum Calcium Level (mg/dL)	8.2 ± 2.1	8.4 ± 2.0	Not significant
Serum Vitamin D Level (ng/mL)	15.2 ± 4.2	27.5 ± 8.1	< 0.0001
Serum Osteocalcin (ng/L)	4.3 ± 1.7	5.6 ± 2.0	< 0.0001
BMD Score			
T-score ≥ - 1	20 (13.3)	26 (17.3)	< 0.0001
T-core between -1 and – 2.5	52 (34.6)	99 (66.0)	
T-score ≤ -2.5	78 (52.0)	25 (16.6)	

## Discussion

In 2010, Alzheimer’s disease was prevalent in around 6% of the world’s population, with 58% of the total Alzheimer’s population belonging to low-middle income countries, and was expected to rise in the years to come [7]. One of the modifiable risk factors identified to the early development of AD is BMD. Our results concluded that a total of 130/150 (86.7%) Alzheimer’s patients presented with low bone mineral density, with 78/150 (52%) being severely affected and discovered to have co-existent osteoporosis (T-score ≤ -2.5).

Our study showed significantly lower vitamin D levels in the Alzheimer’s population (15.2 ng/ml) as compared to the control group (27.5 ng/ml). In a meta-analysis conducted in 2012, it was concluded that serum vitamin D concentrations in AD patients lay 1.4 standard deviations below healthy individuals, labeling it as a potential biomarker for AD and a contributor to declining cognitive function [8-9]. Vitamin D deficiency leads to an increase in the PTH levels, which is further implicated in hastening the decline in mini-mental status scores [10]. Several mechanisms have been proposed but no clear understanding of its pathophysiology has yet been achieved [11]. It is speculated that both the disorders share a common etiology; abnormally expressed amyloid depositions in the brain can cause AD, whereas when deposited in the bone, they interfere with the receptor activator of nuclear factor-kappa-Β ligand (RANKL) signaling cascade leading to an increase in osteoclastic activity thereby, decreasing the BMD and leading to the development of osteoporosis [12].

Similar to our findings, a study conducted in China established a link between osteoporosis and the development of AD; subjects in the lowest quartile of BMD were shown to carry a two-fold increased risk of progression to AD as compared to the healthy population [13]. According to our study, 86.7% of the Alzheimer’s population presented with low BMD but a significant 82.6% of the control population was also discovered to have low BMD. This can be explained by the high prevalence of osteopenia in the Asian population. In a study based in Tehran, it was discovered that regardless of the age, their population had peak bone mass lower than that of the equivalent European or American population [14]. Studies conducted in Pakistan have also shown that the majority of the population, including both men and women, tend to have low BMD [15-16]. However, as per our study, Alzheimer’s population is affected in a greater proportion and much more severe intensity with the majority lying under a T-score of -2.5. In our study, 52% of the Alzheimer’s population was found to have concomitant osteoporosis as compared to 16.6% of the control population. This strongly associates the two comorbid together suggesting an intrinsic link between the pathogenesis of both diseases. With the establishment of this relation, Alzheimer’s patients should be offered dual-energy X-ray absorptiometry (DEXA) scanning to screen for osteoporosis along with appropriate management, which could improve the outcomes of Alzheimer’s progression as it shares the same risk factors. Vitamin D supplementation has been proposed to prevent neurodegeneration in these patients, but further studies are still required to collect sufficient data to comment on its efficacy [17]. In a recent study, the use of vitamin D binding proteins (DBPs), which have been shown to suppress amyloid-related pathology in the brain, has been proposed to slow down the progression of cognitive decline in AD [18-19].

To the best of our knowledge, this is the first study that studies the association between bone health and Alzheimer's disease. However, the study has its limitation as well. First, since the study was at a single institute, sample size diversity was reduced. Second, because the study was case-control in nature, a causal relationship between BMD and AD cannot be established.

## Conclusions

Alzheimer’s disease is associated with poor bone health as compared to the general population of the same age. Bone health can be an important parameter to screen patients at risk of Alzheimer’s disease. The management of Alzheimer’s disease should include a regular assessment of bone health, and the treatment plan should include therapies to improve bone health. Further large-scale prospective studies are needed to establish bone health as a risk factor for Alzheimer's disease.
